# Supplementary material for: Effect of intrapulmonary percussive ventilation on intensive care unit length of stay, the incidence of pneumonia and gas exchange in critically ill patients: A systematic review
Source: PLoS One. 2021 Jul 28;16(7):e0255005. doi: 10.1371/journal.pone.0255005 (PMC8318278; doi:10.1371/journal.pone.0255005)
Supplement: S1 Table — (DOCX) [file pone.0255005.s002.docx]

S1 Table: Search Strategy

| Database: Ovid MEDLINE(R) ALL <1946 to May 20, 2019>  Search Strategy:  --------------------------------------------------------------------------------  1 Critical Illness/ (25727)  2 critical illness.mp. (29872)  3 Critically ill.mp. (40411)  4 acute* ill*.mp. (8109)  5 Critical Care/ (48920)  6 critical care.mp. (65945)  7 Life Support Care/ (7600)  8 life support care.mp. (7789)  9 Intensive Care Units/ (49650)  10 intensive care unit*.mp. (132452)  11 Critical Care/ (48920)  12 intensive care.mp. (158633)  13 ICU.mp. (49978)  14 high dependency unit*.mp. (614)  15 HDU.mp. (301)  16 1 or 2 or 3 or 4 or 5 or 6 or 7 or 8 or 9 or 10 or 11 or 12 or 13 or 14 or 15 (241237)  17 Ventilators, Mechanical/ (8516)  18 mechanical ventilators.mp. (434)  19 mechanical* ventilat*.mp. (46528)  20 pulmonary ventilator.mp. (12)  21 Tracheostomy/ (6974)  22 tracheostomy.mp. (14372)  23 tracheostom*.mp. (15334)  24 pulmonary complications.mp. (9315)  25 Pneumonia/ (45386)  26 pneumonia.mp. (143144)  27 Pulmonary Atelectasis/ (6350)  28 atelectasis.mp. (10702)  29 Pulmonary Disease, Chronic Obstructive/ (35789)  30 chronic obstructive pulmonary disease.mp. (43996)  31 COPD.mp. (42124)  32 Bronchiectasis/ (7727)  33 bronchiectasis.mp. (11858)  34 Cystic Fibrosis/ (33697)  35 cystic fibrosis.mp. (48440)  36 Respiratory Insufficiency/ (30738)  37 respiratory insufficiency.mp. (34643)  38 respiratory failure.mp. (28495)  39 Asthma/ (120415)  40 asthma.mp. (166938)  41 Bronchitis/ (20337)  42 bronchitis.mp. (31829)  43 hyper?secretion.mp. (8611)  44 postoperative .mp. (745756)  45 post surgery.mp. (8457)  46 Neuromuscular Diseases/ (9898)  47 neuromuscular disease.mp. (3313)  48 Muscular Dystrophies/ (13575)  49 Burns/ (43072)  50 burn*.mp. (110890)  51 Burns, Inhalation/ (1080)  52 Smoke Inhalation Injury/ (1210)  53 smoke inhalation.mp. (2133)  54 smoke inhalation injury.mp. (1334)  55 17 or 18 or 19 or 20 or 21 or 22 or 23 or 24 or 25 or 26 or 27 or 28 or 29 or 30 or 31 or 32 or 33 or 34 or 35 or 36 or 37 or 38 or 39 or 40 or 41 or 42 or 43 or 44 or 45 or 46 or 47 or 48 or 49 or 50 or 51 or 52 or 53 or 54 (1391479)  56 16 or 55 (1570702)  57 intrapulmonary percussive ventilation.mp. (70)  58 intrapulmonary percussive ventilator.mp. (8)  59 IPV.mp. (5607)  60 Percussionaire.mp. (15)  61 Percussionator.mp. (2)  62 Metaneb.mp. (5)  63 Metatherapy.mp. (1)  64 Impulsator.mp. (15)  65 IMP2 ventilator.mp. (1)  66 57 or 58 or 59 or 60 or 61 or 62 or 63 or 64 or 65 (5665)  67 "Length of Stay"/ (81479)  68 length of stay.mp. (108466)  69 LOS.mp. (70317)  70 (hospital adj5 stay).mp. [mp=title, abstract, original title, name of substance word, subject heading word, floating sub-heading word, keyword heading word, organism supplementary concept word, protocol supplementary concept word, rare disease supplementary concept word, unique identifier, synonyms] (84755)  71 ICU stay.mp. (6602)  72 Extubate*.mp. (3801)  73 Oxygen/ (159593)  74 oxygen*.mp. (682383)  75 (oxygen adj3 saturation).mp. [mp=title, abstract, original title, name of substance word, subject heading word, floating sub-heading word, keyword heading word, organism supplementary concept word, protocol supplementary concept word, rare disease supplementary concept word, unique identifier, synonyms] (25022)  76 gas* exchange.mp. (26058)  77 SpO2.mp. (4381)  78 PaO2.mp. (11412)  79 Hypoxia/ (61857)  80 hypoxia.mp. (148323)  81 hypoxemia.mp. (14683)  82 hypoxemia.mp. (14683)  83 Hypercapnia/ (8330)  84 hypercapni*.mp. (14760)  85 Acidosis, Respiratory/ (2510)  86 respiratory acidosis.mp. (2028)  87 lung function.mp. (31326)  88 air flow.mp. (3985)  89 airway secretion*.mp. (803)  90 secretion clearance.mp. (184)  91 sputum clearance.mp. (81)  92 mucus clearance.mp. (380)  93 Mucociliary Clearance/ (2404)  94 mucociliary clearance.mp. (4024)  95 (secretion adj3 management).mp. [mp=title, abstract, original title, name of substance word, subject heading word, floating sub-heading word, keyword heading word, organism supplementary concept word, protocol supplementary concept word, rare disease supplementary concept word, unique identifier, synonyms] (95)  96 67 or 68 or 69 or 70 or 71 or 72 or 73 or 74 or 75 or 76 or 77 or 78 or 79 or 80 or 81 or 82 or 83 or 84 or 85 or 86 or 87 or 88 or 89 or 90 or 91 or 92 or 93 or 94 or 95 (1052921)  97 56 and 66 and 96 (58)  98 limit 97 to (humans and yr="1979 -Current") (52)  *************************** |
| --- |
