# Supplementary material for: Effect of intrapulmonary percussive ventilation on intensive care unit length of stay, the incidence of pneumonia and gas exchange in critically ill patients: A systematic review
Source: PLoS One. 2021 Jul 28;16(7):e0255005. doi: 10.1371/journal.pone.0255005 (PMC8318278; doi:10.1371/journal.pone.0255005)
Supplement: S1 File — (DOCX) [file pone.0255005.s003.docx]

**Meta-analysis**

**Data analysis**

Data from three studies with similar study design(1–3) (Antonaglia et al. 2006, Vargas et al. 2005, Huynh et al. 2019) were assessed for their effect size by calculating their confidence interval (95% CI), standardised mean differences, and odds ratio by using the random-effects model. Pooled differences in treatment effects of IPV and CPT (or Control) were performed using the *meta-package* (version 4.10-0. 2015) in R (version 3.6.0, R Foundation for Statistical Computing, Vienna, Austria, URL). Since most studies reported LOS data as medians due to non-normality, studies reporting means were converted to median to reduce bias, and the pooled median difference was reported in this meta-analysis (4). The variances of all the studies, regardless of reporting mean [SD] or median [IQR], were calculated by estimating the probability density function of the underlying distribution by the quantile estimation method at the study level using the *metamedian* package for R (version 0.1.5, 2020)(5). The pooled median difference for ICU-LOS and the pooled odds ratio (OR) for pneumonia incidences were calculated using a random-effects model. Inverse variance weighting was used for pooling. As the number of included studies in each meta-analysis was small, and the sample sizes varied with each study, a random-effects meta-analysis was performed using the Hartung-Knapp-Sidik-Johnson method(6). Between-study variance (τ^2^) was estimated using the Sidik-Jonkman method. Ninety-five percent (95%) confidence interval of the pooled estimates were also reported. *I*^2^ and τ^2^ were used as measures for studies heterogeneity. While *I*^2^ is related to consistency between the study results, τ^2^ reflects the between-study variation. Prediction intervals were also reported and represent the high probable values for the true treatment effects in setting similar to the studies(7). Sensitivity analysis was also performed using the 'leave-one-out' method, where the study results were pooled after leaving each study out.

Due to the small number of studies in each meta-analysis, assessment of publication bias by funnel plot were not performed(8). However, evidential values of true treatment effects were examined using *P*-curves(9). *P*-curve allows one to draw inferences of the distribution of the *P*-values that were less than 0.05 in the included studies: a uniform distribution indicates lack of treatment effect, a strong positively skewed distribution (more studies with small *P*-values) suggests strong evidence of treatment effect, and a negatively skewed distribution indicates possible P-hacking(9). All data were reported as mean [SD] or median [IQR], where appropriate or available. Statistical results were reported or effect size [95% confidence interval (CI)]. *P* - value was not reported for the statistical test, but instead, mean or median [95% confidence interval (CI)] were reported(10,11).

**Outcome measures**

**ICU Length of stay**

The meta-analysis comparing the IPV group to the control group of these three studies demonstrated a beneficial effect in terms of ICU-LOS, where the median ICU-LOS was shorter by 1 to 2 days on average and resulted in an overall shorter pooled median difference (median difference = -1.71 [95%CI: -3.03, -0.39] days) (Figure 1s). The heterogeneity among the studies was substantial (*I*^2^ = 96% [92%, 98%], and τ^2^ = 0.27, *P* < 0.01). The pooled 95%CI of the prediction interval was wide, from -9.36 to 5.93 days (Figure 1s) and the estimated probability of having a null treatment effect (ICU-LOS) or an effect of greater than zero was 11.5% if one of these studies were to be repeated (Figure 2s). The *P*-curve analysis provided evidential value for the positive effects of IPV in reducing ICU-LOS (Figure 2s).

Sensitivity analysis of treatment effects size and heterogeneity (*I*^2^) using the "leave-one-out" method showed that the direction of the effect and effect size were similar to the pooled effect size obtained from the three studies (Figure 3s). Omitting the Vargas et al. (2005) study resulted in a slight reduction in pooled ICU-LOS, i.e., reducing the length of stay by -2.0 days [-2.26, -1.77], and the heterogeneity became minimal (I^2^ = 0). In contrast, omitting the studies by Huynh et al. (2019) and Antonaglia et al. (2006) did not alter the treatment effect significantly (median differences were -1.55 and -1.57 respectively, and heterogeneity remained substantial (*I*^2^ > 0.97).

**Incidence of pneumonia**

Recalculation of the results based on the reported incidence rate did not demonstrate any evidence of a difference between the two groups (Figure 4s). There was also no evidence to support that there was a difference in the pooled incidence rate of pneumonia between the IPV and control groups (Odds ratio: 0.52 [0.09; 2.88]) (Figure 4s).


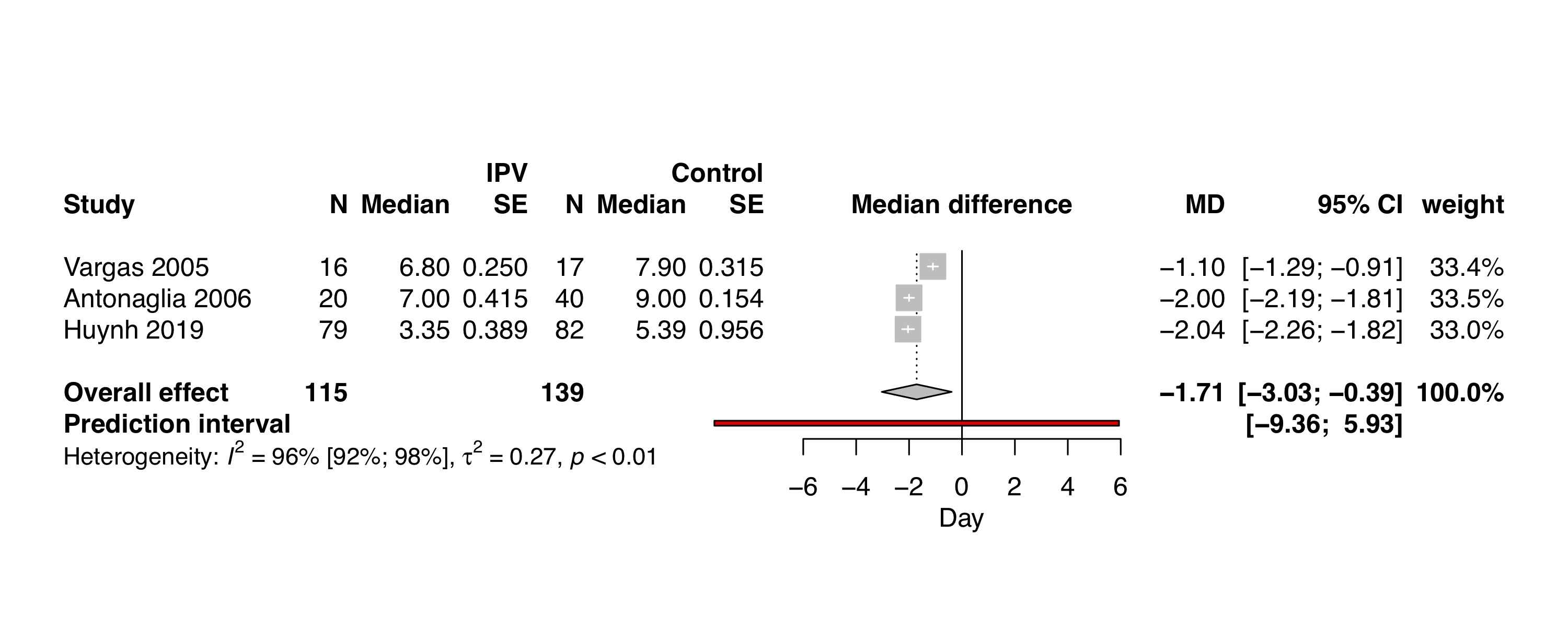


Figure 1s. Forest plot representing length of stay data from three studies. The overall median difference with confidence interval is favouring IPV intervention. The heterogeneity and variance among the studies are represented by *I*^2^ and τ^2^, respectively.

N: number of patients, SE: standard error, MD: Median difference


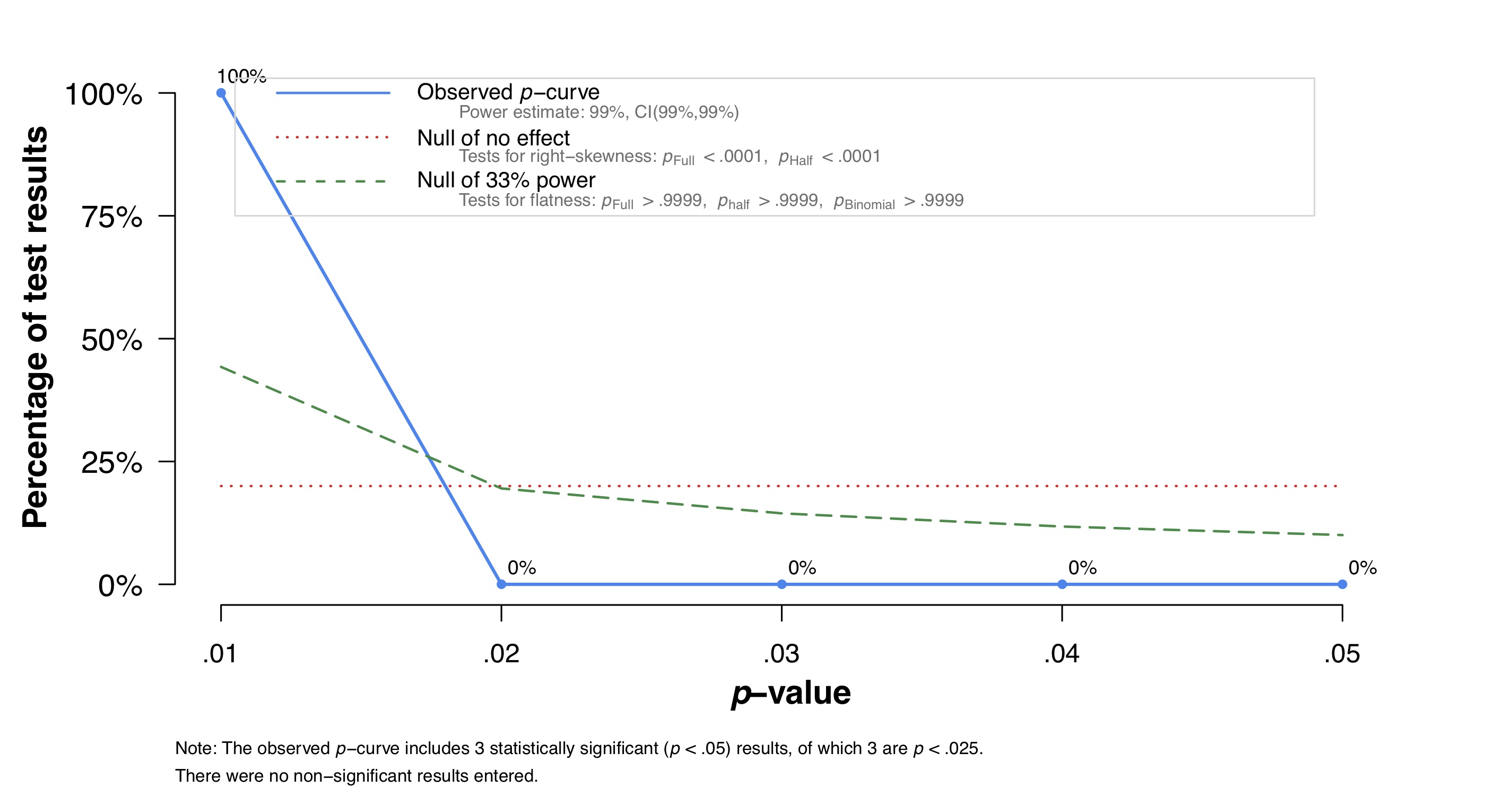


Figure 2s. *P*-curve shows the overall observed *P*-value of all the included studies. The *P*-value of the includes studies are statistically significant (*P* < 0.05)


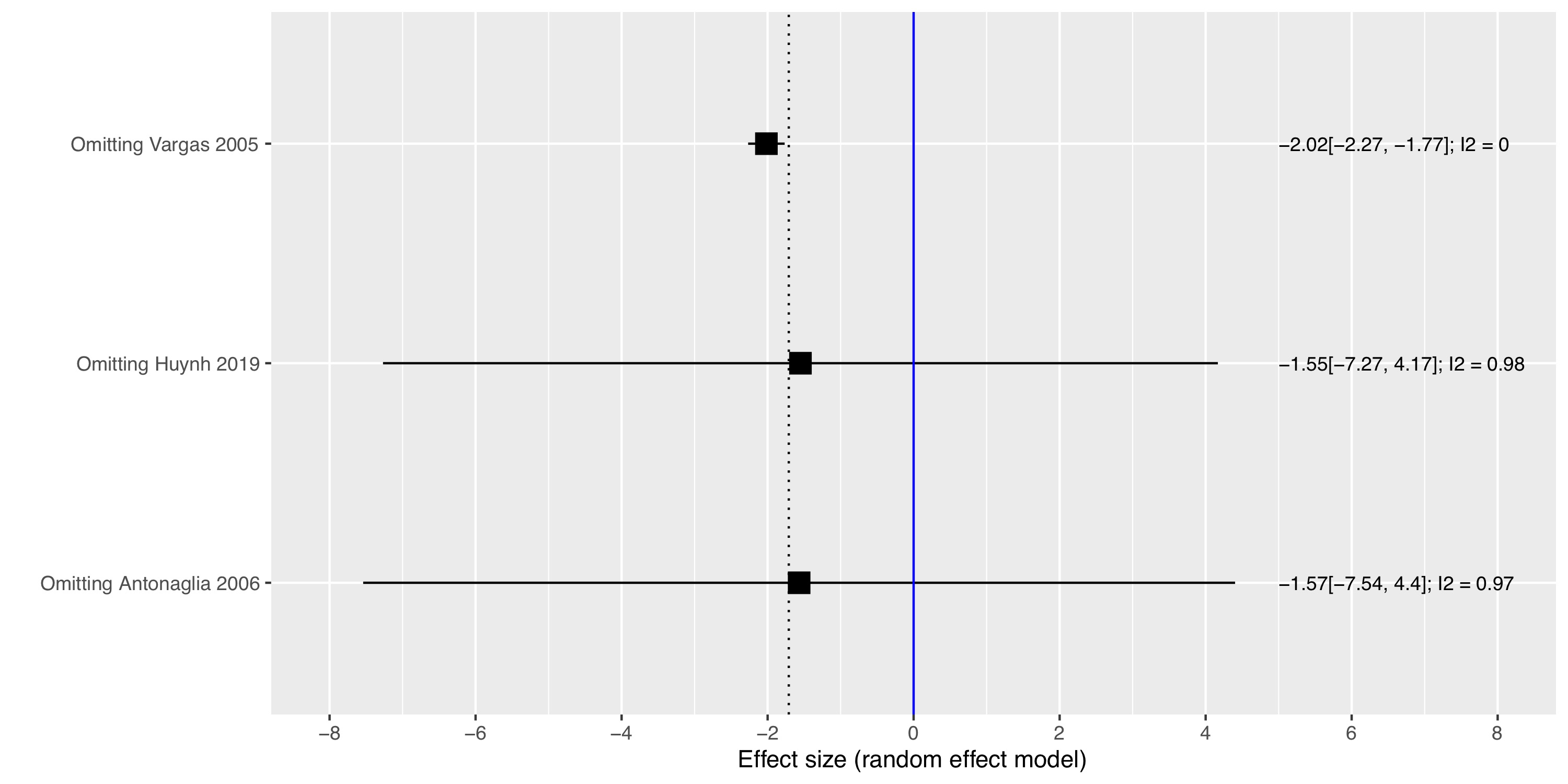


Figure 3s: Random-Effects model for ICU-LOS data. The effect-size is measured by omitting one study at a time using random effect model. Omitting study by Vargas et al. (2005) significantly reduces the heterogeneity score among the studies (*I^2^* = 0) whereas this was not observed when the other studies were omitted.


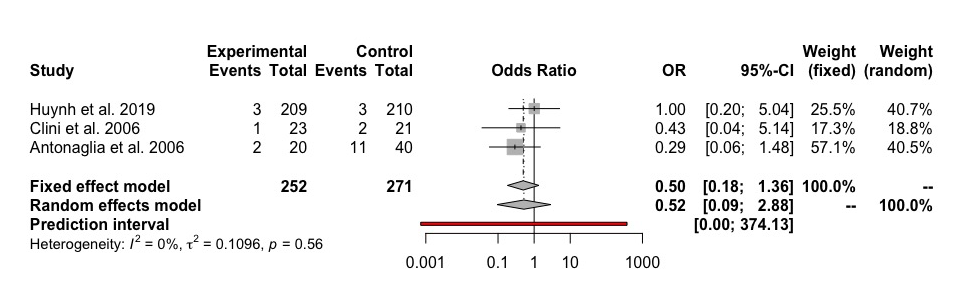


Figure 4s. Forest plot representing the incidence of pneumonia. The heterogeneity and variance within the studies are represented by *I*^2^ and τ^2,^ respectively, OR: odds ratio

**References**

1. Antonaglia V, Lucangelo U, Zin WA, Peratoner A, Simoni LD, Capitanio G, et al. Intrapulmonary percussive ventilation improves the outcome of patients with acute exacerbation of chronic obstructive pulmonary disease using a helmet&ast; Crit Care Med. 2006;34(12):2940–5.

2. Vargas F, Bui HN, Boyer A, Salmi LR, Gbikpi-Benissan G, Guenard H, et al. Intrapulmonary percussive ventilation in acute exacerbations of COPD patients with mild respiratory acidosis: a randomized controlled trial [ISRCTN17802078]. Crit Care. 2005;9(4):R382.

3. Huynh TT, Liesching TN, Cereda M, Lei Y, Frazer MJ, Nahouraii MR, et al. Efficacy of Oscillation and Lung Expansion in Reducing Postoperative Pulmonary Complication. J Am Coll Surgeons. 2019;229(5):458-466.e1.

4. Wan X, Wang W, Liu J, Tong T. Estimating the sample mean and standard deviation from the sample size, median, range and/or interquartile range. Bmc Med Res Methodol. 2014;14(1):135.

5. McGrath S, Sohn H, Steele R, Benedetti A. Meta‐analysis of the difference of medians. Biometrical J. 2020;62(1):69–98.

6. IntHout J, Ioannidis JP, Borm GF. The Hartung-Knapp-Sidik-Jonkman method for random effects meta-analysis is straightforward and considerably outperforms the standard DerSimonian-Laird method. Bmc Med Res Methodol. 2014;14(1):25.

7. Higgins JPT, Thompson SG, Spiegelhalter DJ. A re‐evaluation of random‐effects meta‐analysis. J Royal Statistical Soc Ser Statistics Soc. 2009;172(1):137–59.

8. Lau J, Ioannidis JPA, Terrin N, Schmid CH, Olkin I. The case of the misleading funnel plot. Bmj. 2006;333(7568):597–600.

9. Simonsohn U, Nelson LD, Simmons JP. P-curve_ A Key to The File Drawer. Journal of Experimental Psychology: *General* 2014; 143: 534-54710. Harrington D, D’Agostino RB, Gatsonis C, Hogan JW, Hunter DJ, Normand S-LT, et al. New Guidelines for Statistical Reporting in the Journal. New Engl J Med. 2019;381(3):285–6.

11. Wasserstein RL, Lazar NA. The ASA’s Statement on p-Values: Context, Process, and Purpose. Am Statistician. 2016;70(2):129–33.
