# Supplementary material for: Effect of intrapulmonary percussive ventilation on intensive care unit length of stay, the incidence of pneumonia and gas exchange in critically ill patients: A systematic review
Source: PLoS One. 2021 Jul 28;16(7):e0255005. doi: 10.1371/journal.pone.0255005 (PMC8318278; doi:10.1371/journal.pone.0255005)
Supplement: Attachment — Submitted filename: IPPV Syst Rev PONE-D-21-11360_reviewer.pdf [file pone.0255005.s006.pdf]

PLOS ONE

Effect of intrapulmonary percussive ventilation on intensive care unit length of stay, incidence of pneumonia and gas exchange in critically ill patients: a systematic review

--Manuscript Draft--

**Manuscript Number:**	PONE-D-21-11360
**Article Type:**	Research Article
**Full Title:**	Effect of intrapulmonary percussive ventilation on intensive care unit length of stay, incidence of pneumonia and gas exchange in critically ill patients: a systematic review
**Short Title:**	Intrapulmonary percussive ventilation in critical care: a systematic review
**Corresponding Author:**	Anwar Hassan, MHSc (Cardio Pulm)<br>Nepean Blue Mountains Local Health District<br>Glenmore Park, New South Wales AUSTRALIA
**Keywords:**	Intrapulmonary percussive ventilation; percussive ventilation; chest physiotherapy; critical care; critically ill; intensive care
**Abstract:**	Background<br><br>Pulmonary complications such as pneumonia, pulmonary atelectasis, and subsequent respiratory failure leading to ventilatory support are a common occurrence in critically ill patients. Intrapulmonary percussive ventilation is used to improve gas exchange and promote airway clearance in these patients. The current evidence regarding the effectiveness of intrapulmonary percussive ventilation in critical care setting remains unclear. This systematic review aims to summarise the evidence of the effectiveness of intrapulmonary percussive ventilation on intensive care unit length of stay (ICU-LOS) and various respiratory outcomes in critically ill patients.<br><br>Research question<br><br>In critically ill patients in ICU, is intrapulmonary percussive ventilation effective in improving respiratory outcomes and reducing intensive care unit length of stay.<br><br>Study design and Methods<br><br>A systematic search of intrapulmonary percussive ventilation in intensive care unit (ICU) was performed on five databases from 1979 to 2019. Studies were considered for inclusion if they evaluated the effectiveness of IPV in patients aged ≥16 years receiving invasive or non-invasive ventilation or breathing spontaneously in critical care or high dependency unit. Study titles and abstracts were screened, followed by data extraction by full-text review. Outcomes of interest were summarised narratively.<br><br>Results<br><br>Out of 306 studies, seven studies (630 patients) met the eligibility criteria. Results of the included studies showed that intrapulmonary percussive ventilation may reduce ICU-LOS. Evidence also showed that IPV intervention might improve gas exchange and reduce respiratory rate. Substantial heterogeneity and variance were noted among the included studies; hence a meta-analysis could not be included in this review.<br><br>Interpretation<br><br>The findings of this review provide some evidence that IPV intervention may reduce ICU-LOS, improve gas exchange and reduce respiratory rate in critically ill patients.
**Order of Authors:**	Anwar Hassan, MHSc (Cardio Pulm)
	William Lai
	Jennifer Alison
	Stephen Huang

Powered by Editorial Manager® and ProduXion Manager® from Aries Systems Corporation

	Maree Milross
**Additional Information:**	
**Question**	**Response**
**Financial Disclosure**<br><br>Enter a financial disclosure statement that describes the sources of funding for the work included in this submission. Review the submission guidelines for detailed requirements. View published research articles from *PLOS ONE* for specific examples.<br><br>This statement is required for submission and **will appear in the published article** if the submission is accepted. Please make sure it is accurate.<br><br>**Unfunded studies**<br>Enter: *The author(s) received no specific funding for this work.*<br><br>**Funded studies**<br>Enter a statement with the following details:<br>• Initials of the authors who received each award<br>• Grant numbers awarded to each author<br>• The full name of each funder<br>• URL of each funder website<br>• Did the sponsors or funders play any role in the study design, data collection and analysis, decision to publish, or preparation of the manuscript?<br>• **NO** - Include this sentence at the end of your statement: *The funders had no role in study design, data collection and analysis, decision to publish, or preparation of the manuscript.*<br>• **YES** - Specify the role(s) played.<br><br>* typeset	The authors received no specific funding for this work
**Competing Interests**<br><br>Use the instructions below to enter a competing interest statement for this submission. On behalf of all authors, disclose any competing interests that could be perceived to bias this work—acknowledging all financial support and any other relevant financial or non-financial competing interests.	The authors have declared that no competing interest exist

This statement is required for submission and **will appear in the published article** if the submission is accepted. Please make sure it is accurate and that any funding sources listed in your Funding Information later in the submission form are also declared in your Financial Disclosure statement.<br><br>View published research articles from *PLOS ONE* for specific examples.<br><br>**NO authors have competing interests**<br><br>Enter: *The authors have declared that no competing interests exist.*<br><br>**Authors with competing interests**<br><br>Enter competing interest details beginning with this statement:<br><br>*I have read the journal's policy and the authors of this manuscript have the following competing interests: [insert competing interests here]*<br><br>* typeset	
**Ethics Statement**<br><br>Enter an ethics statement for this submission. This statement is required if the study involved:<br><br>• Human participants<br>• Human specimens or tissue<br>• Vertebrate animals or cephalopods<br>• Vertebrate embryos or tissues<br>• Field research<br><br>Write "N/A" if the submission does not require an ethics statement.<br><br>General guidance is provided below. Consult the submission guidelines for detailed instructions. **Make sure that all**	Ethics was not required for conducting this Systematic review

Powered by Editorial Manager® and ProduXion Manager® from Aries Systems Corporation

information entered here is included in the Methods section of the manuscript.<br><br>**Format for specific study types**<br><br>**Human Subject Research (involving human participants and/or tissue)**<br>• Give the name of the institutional review board or ethics committee that approved the study<br>• Include the approval number and/or a statement indicating approval of this research<br>• Indicate the form of consent obtained (written/oral) or the reason that consent was not obtained (e.g. the data were analyzed anonymously)<br><br>**Animal Research (involving vertebrate animals, embryos or tissues)**<br>• Provide the name of the Institutional Animal Care and Use Committee (IACUC) or other relevant ethics board that reviewed the study protocol, and indicate whether they approved this research or granted a formal waiver of ethical approval<br>• Include an approval number if one was obtained<br>• If the study involved *non-human primates*, add *additional details* about animal welfare and steps taken to ameliorate suffering<br>• If anesthesia, euthanasia, or any kind of animal sacrifice is part of the study, include briefly which substances and/or methods were applied<br><br>**Field Research**<br><br>Include the following details if this study involves the collection of plant, animal, or other materials from a natural setting:<br>• Field permit number<br>• Name of the institution or relevant body that granted permission	
**Data Availability**<br><br>Authors are required to make all data underlying the findings described fully available, without restriction, and from the time of publication. PLOS allows rare exceptions to address legal and ethical	Yes - all data are fully available without restriction

concerns. See the PLOS Data Policy and FAQ for detailed information.<br><br>A Data Availability Statement describing where the data can be found is required at submission. Your answers to this question constitute the Data Availability Statement and **will be published in the article**, if accepted.<br><br>**Important:** Stating 'data available on request from the author' is not sufficient. If your data are only available upon request, select 'No' for the first question and explain your exceptional situation in the text box.<br><br>Do the authors confirm that all data underlying the findings described in their manuscript are fully available without restriction?	
**Describe where the data may be found in full sentences. If you are copying our sample text, replace any instances of XXX with the appropriate details.**<br><br>• If the data are **held or will be held in a public repository**, include URLs, accession numbers or DOIs. If this information will only be available after acceptance, indicate this by ticking the box below. For example: *All XXX files are available from the XXX database (accession number(s) XXX, XXX.*).<br>• If the data are all contained **within the manuscript and/or Supporting Information files**, enter the following: *All relevant data are within the manuscript and its Supporting Information files.*<br>• If neither of these applies but you are able to provide **details of access elsewhere**, with or without limitations, please do so. For example:<br><br>*Data cannot be shared publicly because of [XXX]. Data are available from the XXX Institutional Data Access / Ethics Committee (contact via XXX) for researchers who meet the criteria for access to confidential data.*	It is a systematic review so the search results and results of data extraction can be provided upon acceptance of the manuscript on request.

The data underlying the results presented in the study are available from (include the name of the third party and contact information or URL).<br>• This text is appropriate if the data are owned by a third party and authors do not have permission to share the data.<br><br>* typeset	
Additional data availability information:	Tick here if your circumstances are not covered by the questions above and you need the journal's help to make your data available.

Powered by Editorial Manager® and ProduXion Manager® from Aries Systems Corporation

Manuscript

Click here to access/download;Manuscript;IPV in Critical care_Systematic review_Manuscript_AHassan.docx

TITLE

Effect of intrapulmonary percussive ventilation on intensive care unit length of stay, incidence of pneumonia and gas exchange in critically ill patients: a systematic review

Anwar Hassan[1,2], William Lai[1], Jennifer Alison[2], Stephen Huang[3,4], and Maree Milross[2]

AUTHORS

Anwar Hassan, MHSc (CardioPulm) (First author and Contact person)
Physiotherapy Department, Nepean Hospital[1]
Sydney School of Health Sciences, Faculty of Medicine and Health, The University of Sydney[2]
anwarul.hassan@health.nsw.gov.au
ORCID iD: 0000-0003-2000-5434

William Lai, BPhty
Physiotherapy Department, Nepean Hospital[1]
william.lai@health.nsw.gov.au
ORCID iD: 0000-0001-9932-167X

Prof Jennifer Alison, PhD
Sydney School of Health Sciences, Faculty of Medicine and Health, The University of Sydney[2]
jennifer.alison@sydney.edu.au
ORCID iD: 0000-0002-2011-4756

Prof Stephen Huang, PhD
Intensive Care Unit, Nepean Hospital[3]
Department of Medicine, The University of Sydney[4]
stephen.huang@sydney.edu.au
ORCID iD: 0000-0003-2518-0506

Dr. Maree Milross, PhD

Sydney School of Health Sciences, Faculty of Medicine and Health, The University of Sydney[2]
maree.milross@sydney.edu.au
ORCID iD: 0000-0001-8587-3895

Abbreviations: COPD = chronic obstructive pulmonary disease; CPT = chest physiotherapy; FiO2 = fraction of inspired oxygen; IPV = intrapulmonary percussive ventilation; ICU-LOS = intensive care unit length of stay; NIV = non-invasive ventilation; $PaCO_2$ = partial pressure of arterial carbon dioxide; PaO_2 = partial pressure of arterial oxygen; SpO2 = saturation of peripheral oxygen

Abstract

Background: Pulmonary complications such as pneumonia, pulmonary atelectasis, and subsequent respiratory failure leading to ventilatory support are a common occurrence in critically ill patients. Intrapulmonary percussive ventilation is used to improve gas exchange and promote airway clearance in these patients. The current evidence regarding the effectiveness of intrapulmonary percussive ventilation in critical care setting remains unclear. This systematic review aims to summarise the evidence of the effectiveness of intrapulmonary percussive ventilation on intensive care unit length of stay (ICU-LOS) and various respiratory outcomes in critically ill patients.

Research question: In critically ill patients in ICU, is intrapulmonary percussive ventilation effective in improving respiratory outcomes and reducing intensive care unit length of stay.

Study design and Methods: A systematic search of intrapulmonary percussive ventilation in intensive care unit (ICU) was performed on five databases from 1979 to 2019. Studies were considered for inclusion if they evaluated the effectiveness of IPV in patients aged ≥16 years receiving invasive or non-invasive ventilation or breathing spontaneously in critical care or high dependency unit. Study titles and abstracts were screened, followed by data extraction by full-text review. Outcomes of interest were summarised narratively.

Results: Out of 306 studies, seven studies (630 patients) met the eligibility criteria. Results of the included studies showed that intrapulmonary percussive ventilation may reduce ICU-LOS. Evidence also showed that IPV intervention might improve gas exchange and reduce respiratory rate. Substantial heterogeneity and variance were noted among the included studies; hence a meta-analysis could not be included in this review.

Interpretation: The findings of this review provide some evidence that IPV intervention may reduce ICU-LOS, improve gas exchange and reduce respiratory rate in critically ill patients.

Keywords: Intrapulmonary percussive ventilation, percussive ventilation, chest physiotherapy, critical care, critically ill, intensive care

Registration: PROSPERO Registration ID: CRD42018115517

Declarations: None

Funding: Authors did not receive any funding for this review

Conflicts of interest: None of the authors (AH, WL, JA, SH, and MM) have any conflict of interest to report.

Introduction

The incidence of pulmonary complications such as pulmonary atelectasis, pneumonia (including ventilator-associated pneumonia), and acute respiratory failure is high in critical care patients(1,2). The incidence of ventilator-associated pneumonia can be as high as 27% amongst mechanically ventilated patients(3). Studies have shown that 16% of critically ill patients have been reported to develop acute respiratory failure, which is associated with prolonged intensive care unit stay, resulting in significantly higher mortality than non-respiratory failure patients(2,4–7). Increased morbidity and mortality contribute to the burden on the health care system and lead to poor health-related outcomes(6–8). Multimodal physiotherapy plays a role in the management of these critically ill patients(9). Respiratory physiotherapy interventions aim to promote airway secretion clearance, increase alveolar recruitment, minimise pulmonary shunting, and optimise ventilation and perfusion (V/Q) matching(10,11). In addition to conventional chest physiotherapy (CPT)(12), intrapulmonary percussive ventilation (IPV) is used in patients with underlying pulmonary atelectasis, with or without coexisting excessive airway secretions or respiratory failure(12–15).

IPV is a non-continuous form of high-frequency ventilation delivered by a pneumatic device that provides small bursts of sub-physiological tidal breaths at a frequency of 60 - 600 cycles/minute superimposed on patient's breathing cycle(16–18). In acute care and critical care settings, this intervention is used in a range of patients, from spontaneously breathing patients to those receiving invasive mechanical ventilation(13,14,18). In the last two decades, few studies have reported IPV in the critical care setting to be effective in improving outcomes in patients with an acute exacerbation of chronic obstructive pulmonary disease (COPD), burns, pulmonary atelectasis, and those with post-abdominal or thoracic surgery(14,15,19,20). Despite the available studies, the overall evidence regarding its effectiveness in critical care settings remains unclear. Recently, Reychler and colleagues (2018)(21) summarised the

effectiveness of IPV in chronic lung diseases such as COPD, cystic fibrosis, and bronchiectasis. However, most of the studies reviewed by Reychler and colleagues (2018) included non-critically ill stable patients(21). Also, the review included studies that evaluated the airway clearance and gas exchange properties of IPV which leaves the question regarding the role of IPV in preventing or reversing atelectasis in acutely ill patients unanswered. The objective of this systematic review was to summarise the evidence for the effectiveness of IPV in improving outcomes such as intensive care unit length of stay (ICU-LOS), gas exchange, respiratory rate, incidence of pneumonia and reversing or preventing atelectasis in critical care patients.

Methods

This systematic review followed recommendations from the Preferred Reporting Items for Systematic Reviews and Meta-Analysis (PRISMA)(22). The review protocol was registered in the International Prospective Register of Systematic Reviews (PROSPERO) before conducting the database search (Registration ID: CRD42018115517).

Search strategy

A systematic search of the literature was conducted in two stages. The first stage included database searches on MEDLINE, EMBASE, CINAHL, Web of Science, and PEDro, from 1979 (when IPV was first introduced) to May 2019. The details of search strategy and keywords used are presented in Supplementary Table 1S. The second stage included searching the relevant clinical trial registries (including ClinicalTrials.gov, ANZCTR, WHO, EUCTR, ATS, PROSPERO). A manual search of the reference lists of the included studies were also conducted. An update search in February 2021 did not result in any new studies.

Inclusion and exclusion criteria

Studies were considered for inclusion if they evaluated the effectiveness of IPV in patients aged ≥16 years receiving invasive or non-invasive ventilation (NIV) or breathing spontaneously in critical care unit for acute or acute on chronic impairment of respiratory function. Studies that included stable, non-acutely ill patients in the inpatient, outpatient, or community-based settings were excluded.

Due to the limited number of studies, randomised controlled trials (RCT), quasi-randomised trials, randomised crossover studies, observational studies, comparative studies, experimental designs with random allocation, and retrospective studies were all considered. Studies that reported the effects of IPV, high-frequency ventilation, and high-frequency oscillation where these interventions were primarily used for therapeutic purposes were included, whereas the studies that used these interventions to provide continuous mechanical ventilation were excluded.

Studies that measured ICU-LOS and examined the physiological variables such as changes in the saturation of peripheral oxygen (SpO_2) and partial pressure of arterial oxygen (PaO_2) and partial pressure of arterial carbon dioxide ($PaCO_2$) measured by arterial blood gas analysis were included. Other outcomes such as pulmonary atelectasis and respiratory rate, were also included.

Study review and data extraction

Duplicates were removed using the Covidence® software, followed by a manual search for duplicates. The remaining articles were screened independently by two authors (AH and WL) by reviewing the study titles and abstracts (inter-rater reliability, Kappa 0.84). Authors of the eligible studies with published abstract only or papers with missing or insufficient data were contacted via email for the full-text article or raw data; the study was excluded if no response was obtained within four weeks. Full-text review was conducted by two authors (AH and

WL), and ineligible studies were excluded (Figure 1). In case of disagreement, a third reviewer (MM) independently reviewed the study. Included studies were then used for data extraction and quality assessment for the risk of bias.

Figure 1. PRISMA flow chart

Assessment of quality and risk of bias

The quality and risk of bias were assessed according to the Cochrane Collaboration assessment tool during the data extraction phase using the Covidence® software(23). For all the included literature, the following risk of bias domains was assessed: random sequence generation, allocation sequence concealment, blinding of participants, blinding of the therapist, blinding of outcome assessors, incomplete outcome data, selective outcome reporting, and overall risk of bias. The level of risk of bias was assessed under three categories: 1) high, 2) low, and 3) unclear (Table 1). In addition, the Physiotherapy Evidence Database (PEDro) scale was also used to assess and summarise the study quality (Table 2)(24). PEDro scale allows assessment on ten different domains to determine the study quality. Based on the total PEDro score studies can be categorised into; “poor” (score 0-3), “fair” (score 4-5), “good” (score 6-8) and “excellent” (score 9-10). Overall the studies were found to have moderate risk of bias.

Table 1. Risk of bias

Study	Random sequence generation	Allocation concealment	Blinding of participants and personnel	Blinding of outcome assessors	Incomplete outcome data	Selective reporting	Other bias
Antonaglia et al. (2006)	Low	Low	High	High	Low	Low	Low
Vargas et al. 2005	Low	Low	High	High	Unclear	Unclear	Low
Huynh et al. 2019	Unclear	High	High	High	Unclear	Unclear	High
Clini et al. 2006	Low	Low	High	Low	Low	Low	Low
Dimassi et al. 2011	Low	High	High	Unclear	Low	High	High
Vargas et al. 2009	High	High	High	High	Low	Low	High
Tsuruta et al. 2006	Unclear	High	High	High	Low	Low	High

Table 2. Quality assessment using PEDro scale(24)

Study	Eligibility criteria	Random allocation	Concealed allocation	Groups similar at baseline	Subject blinding	Therapist blinding	Assessor blinding	< 15% dropouts	Intention to treat analysis	Statistical comparison in groups	Measure of variability	Total score
Antonaglia 2006(13)	Y	Y	Y	Y	N	N	N	N	N	Y	Y	5/10
Vargas 2005(15)	Y	Y	Y	Y	N	N	N	Y	N	Y	Y	6/10
Huynh 2019(20)	Y	N	N	N	N	N	N	Y	Y	Y	Y	4/10
Clini 2006(12)	Y	Y	Y	Y	N	N	Y	Y	N	Y	Y	7/10
Dimassi 2011(26)	Y	Y	N	Y	N	N	N	Y	N	Y	Y	5/10
Vargas 2009(25)	Y	N	N	N	N	N	N	Y	N	N	Y	2/10
Tsuruta 2006(14)	Y	N	N	N	Y	N	N	Y	N	N	Y	3/10

Y= Yes, N =No. PEDro score (0 - 3 = poor, 4 - 5 = fair, 6 - 8 = good, 9 - 10 = excellent)

Outcome measures

The outcomes of interest were ICU-LOS, PaO_2, the ratio of the partial pressure of arterial oxygen and fraction of inspired oxygen (PaO_2/FiO_2), $PaCO_2$, respiratory rate, and incidence of pneumonia.

Due to the observed heterogeneities in the study methodology and patient population, a meta-analysis was not included, instead, all the outcomes were summarised narratively.

Results

The database search yielded a total of 306 studies. After removing 68 duplicates, the titles and abstracts were screened for 238 studies. After excluding 199 irrelevant studies, the remaining 39 full-text articles were assessed for their eligibility. The reviewers identified seven studies including 630 patients, that met the eligibility criteria for systematic review (Figure 1). The study characteristics are described in Table 3.

Table 3. Study summary (attached separately)

Study Characteristics

Among the included studies, four studies were RCTs, one was a quasi-RCT with a historical control group, and the remaining two were prospective observational studies. The observational studies did not assign a control group for comparison(14,25). Study sample sizes ranged from 10 patients to 419 patients(14,20). All the studies were conducted in the critical care setting where patients were receiving invasive mechanical ventilation, non-invasive ventilation, or were breathing spontaneously with supplemental oxygen. Patients in one study were postoperative, with some receiving invasive mechanical ventilation while others were spontaneously breathing(20), whereas, in another study, all the patients were receiving mechanical ventilation(14).

Patient characteristics

A total of 630 patients, in 7 included studies, were admitted to an intensive care unit (Table 3). Patient's ages ranged from 18 to 95 years (mean age 63.7 years), with 60% males. One study did not report the mean age(26). Common clinical conditions were post thoracic, aortic or abdominal surgeries(20), acute respiratory failure secondary to COPD(13,15,25), post-extubation respiratory failure(26), pulmonary atelectasis(14) and tracheostomised patients with impaired respiratory function(12).

Intervention

Most common indication to use IPV was to improve gas exchange, promote airway clearance and prevent or reverse pulmonary atelectasis. The treatment application varied among the studies. In most studies, IPV was delivered via a facemask or mouthpiece, whereas for those who were mechanically ventilated, IPV was delivered via an in-line ventilator circuit(14,20). The device used to deliver IPV in the included studies were Percussionator®, IPV1-C® (Percussionaire Corporation, Sandpoint, ID, USA) and Metaneb® (Hill-ROM corporation, USA). All these device work on the same mechanical principles and uses phasitron to deliver similar breath frequency (200 to 300 cycles/minute) and airway pressures. The treatment dosage, such as duration and frequency of sessions per day, varied across the studies. For instance, the duration of a single treatment session ranged from 10 to 30 minutes, and the number of sessions ranged from a single session a day to up to six sessions a day(20). The frequency of delivered breaths remained between 200 to 300 cycles per minute in all the included studies whereas the airway pressure varied from 5 to 35 cmH_2O. Notably, most of the studies did not specify the patient's position during the treatment. IPV intervention was compared to routine CPT or standard medical treatment.

Outcome measures

The common outcomes reported were ICU-LOS, the incidence of pneumonia, changes in PaO_2, PaO_2/FiO_2, $PaCO_2$ and respiratory rate. Less commonly, studies also recorded the incidence of pulmonary atelectasis, changes in diaphragmatic work, duration of mechanical ventilation, need for NIV, hospital length of stay, and mortality. Some of these physiological outcomes (PaO_2, PaO_2/FiO_2, $PaCO_2$ and respiratory rate) were recorded daily before and after the intervention, and also at the time of discharge whereas one study recorded these outcomes at five-day intervals for up to 15 days(12).

ICU length of stay

Among the included studies, three studies reported ICU-LOS(13,15,20). Antonaglia and colleagues (2006)(13) randomly allocated 40 critically ill patients with an acute exacerbation of COPD to the IPV group (n = 20) or standard CPT group (n = 20) where patients in both the groups were treated with NIV. In addition to NIV, patients in the CPT group received standard chest physiotherapy for 25-30 minutes and those in the intervention group received 25-30 minutes of IPV twice a day. This study also included a historical control group (n = 40) for comparison that received standard medical treatment. A significantly shorter ICU-LOS in the IPV group (median = 7 [6, 8] days) than the control group was reported (median = 10 [9, 11] days), median difference -2.0 days (95% CI: -2.19; -1.81 days, $p < 0.01$). Similarly, a multicentre study by Huynh et al. (2019)(20) evaluated the effect of IPV in 419 postoperative (upper abdominal, aortic and thoracic surgery) patients admitted to ICU, where the intervention group (n = 209) received IPV for 10 minutes four to six times a day, and the historical control group (n = 210) received standard CPT mainly in the form of incentive spirometry. Out of 419 patients, the ICU-LOS was reported only for 161

patients (Intervention = 79, Control = 82) where the ICU-LOS was found to be shorter but not statistically significant in the IPV group (IPV: mean 3.3 [3.5] days vs. Control: mean 5.4 [8.7] days, *NS*). Another study by Vargas et al. (2005) investigated the effects of IPV intervention in 33 patients with COPD with acute respiratory failure (15). The intervention group (n = 17) received IPV for 30 minutes twice a day whereas control group (n =16) received standard treatment. Study reported a significant reduction in length of stay in the IPV group compared to the control group (IPV: mean 6.8 [1.0] days vs. Control: 7.9 [1.3] days, $p < 0.05$).

Incidence of pneumonia

Among the included studies, the incidence of pneumonia was reported by three studies(12,13,20). Antonaglia et al. (2006) reported low incidence of pneumonia (IPV: 4 vs. CPT: 2, *NS*) in 40 patients with acute exacerbation of COPD. Although the incidence of pneumonia in the IPV group was half of that of the CPT group, it was not statistically significant(13). Similarly, Huynh et al. (2019) did not find any statistically significant difference in the incidence of pneumonia in 419 patients with upper abdominal and thoracic surgery patients (IPV: 3 vs CPT: 3, *NS*)(20). However, one study reported a significant reduction in the incidence of pneumonia in tracheostomised patients treated with IPV (IPV: 3 vs. CPT: 5, $p < 0.05$)(12).

Gas exchange

a) **PaO_2 and PaO_2/ FiO_2 ratio**

   Six studies (n = 211) reported an increase in oxygenation in the IPV group(12–15,25,26). The increase in oxygenation was recorded as a change from baseline to post-intervention, as PaO_2 and or the PaO_2/ FiO_2 ratio.

The most significant change in PaO_2/FiO_2 ratio was reported by Antonaglia et al. (2006)(13) from admission to discharge (seven days) in patients with COPD admitted to ICU (IPV: 173 [27] to 274 [15], $p < 0.01$ and Control: 181 [29] to 237 [20], $p < 0.01$). Similarly, Clini et al. (2006)(12) also reported an improvement in PaO_2/FiO_2 ratio in 46 patients with tracheostomy randomised to receive either CPT or IPV in addition to CPT, after 15 days of intervention (IPV: 238 [51] to 289 [52], $p < 0.005$, Control: 240 [34] to 255 [38], $p < 0.05$), median difference 21.65 (95% CI: -11.75 to – 55.05, $p < 0.038$). Similarly, Vargas et al. (2005)(15) found a significant increase in PaO_2 in patients with COPD who received IPV intervention (56.9 [3] to 61 [0.8] mmHg, $p < 0.05$). These findings of increased oxygenation (PaO_2/FiO_2 ratio and PaO_2) in the IPV group were consistent with the findings of two observational studies(14,25). In contrast, one small study of 17 post-extubation patients, who received IPV intervention and NIV in random order, reported no significant change in the PaO_2/FiO_2 ratio(26).

b) Change in $PaCO_2$

A total of six studies evaluated a change in the $PaCO_2$ levels(12–15,25,26). Antonaglia et al. (2006) recorded a significant reduction in $PaCO_2$ levels in patients with COPD in IPV and CPT group (IPV: 79 [7] to 58 [5.4], $p < 0.01$, Control: 80 [6.5] to 64 [5.2] mmHg, $p < 0.01$)(13). Similar findings were reported by Vargas et al (2005) where a significant reduction in the $PaCO_2$ levels were seen in the IPV group (IPV: 57.6 [4.5] to 53.5 [2.3] mmHg, $p < 0.05$)(15). The study did not report any data for the control group for comparison. In another small study by Vargas and colleagues (2009) in 25

patients (with no control group) with acute exacerbation of COPD found a reduction in $PaCO_2$ (IPV: 55.1 [3.7] to 52.5 [2.2] mmHg, $p < 0.05$)(25). A small, non-significant reduction in $PaCO_2$ levels were also reported by Clini et al. (2006) in the IPV group without any change in the control group(12).

Respiratory rate

Among the included studies, four studies evaluated effects of IPV intervention on patient's respiratory rate (RR)(13,15,25,26) . Vargas and colleagues (2005)(15) found a significant reduction in RR in COPD patients in the IPV group (36 [2] to 31 [2] breaths per minute, $p < 0.05$) with no change in the control group. In another study in 25 COPD patients, Vargas et al. (2009) reported a small reduction in RR (IPV: 22.6 [2.3] to 21.4 [1.7] breaths per minute, $p < 0.05$)(25). Similarly, a small but significant reduction in respiratory rate was observed by Dimassi et al. (2011) in 17 post-extubation patients (23, [19-27] to 22, [17-24] breaths per minute, $p < 0.01$)(26). In contrast, reports from Antonaglia et al. (2006) study did not demonstrate any significant change in RR(13). Overall, three out of four studies reported a small but significant reduction in respiratory rate post IPV intervention. This small change does not seem to be clinically relevant.

Adverse events and tolerance

None of the studies reported any major adverse events related to IPV intervention(12–14,20,26). Vargas et al. (2005) reported a single incidence of haemoptysis in one patient, unrelated to IPV intervention(15). One study did not report on adverse events(25). Four studies(12,13,15,26), based on their observational findings,

mentioned that the IPV intervention was well-tolerated, none of the studies asked specific questions pertaining to IPV tolerance. A recent multicentre study found one minor episode of IPV intolerance, which resolved quickly, and therapy was resumed 8 hours later (20).

Discussion

This systematic review synthesised the evidence of the effectiveness of IPV intervention in critical care patients. The findings of this review provide some evidence that IPV intervention may reduce ICU-LOS, improve gas exchange and reduce respiratory rate in critically ill patients. Similar findings in patients with chronic lung disease have been reported in another systematic review including 12 studies (278 patients) in patients with acute exacerbation of COPD, cystic fibrosis and bronchiectasis in a range of clinical settings (21). This review by Reychler et al. (2018) found that the use of IPV intervention in patients hospitalised with an acute exacerbation of COPD (n = 178) improved gas exchange (PaO_2 and $PaCO_2$) compared to routine chest physiotherapy and might reduce hospital LOS. Our systematic review is the first one to summarise the effectiveness of IPV intervention in critical care population. However, the findings of our systematic review should be viewed with caution since there were various methodological (study design, outcome measures), clinical (patient population and application of IPV) and statistical (small sample size and lack of control group) heterogeneities were observed. The overall risk of bias among the included studies was found to be moderate (Table 1 and 2).

Studies that measured the effect of IPV on length of stay demonstrated some beneficial effects, where the median ICU-LOS appeared to be shortened by 1 to 2 days in the IPV group. However, significant heterogeneity was observed among these three studies that

reported on ICU-LOS. Two studies (Vargas et al. 2005 and Antonaglia et al. 2006) included patients with acute exacerbation of COPD whereas, Huynh et al (2019) included upper abdominal and thoracic surgery patients. Huynh et al (2019) found a non-significant reduction in ICU-LOS, but reported a significant reduction in hospital LOS in the IPV group (IPV: 6.78 [4.98] vs. CPT: 8.40 [7.9] days $p < 0.02$). This outcome of hospital LOS, however, should be interpreted with caution as the non-randomised study design and the treatment frequency in the IPV group may introduce some bias. The duration and frequency of IPV intervention also varied among the included studies. The COPD patients in the two studies (Antonaglia et al and Vargas et al 2005) received 30 minutes of IPV sessions two times a day, whereas Huynh et al provided four to six sessions per day lasting 10 minutes each. Due to the heterogeneity, meta-analysis could not be included in this review, however, interestingly, pooling of ICU-LOS data revealed that the magnitude and direction of the effect of IPV in reducing the ICU-LOS was similar in all three studies. A summary of statistical analysis of the ICU-LOS data is attached for further reading (electronic supplementary material).

Studies have reported that IPV improves gas exchange (PaO_2, PaO_2/FiO_2 ratio, and $PaCO_2$) in ventilated and non-ventilated patients(13,14). In this review, six (of the seven) studies measured PaO_2 or PaO_2/FiO_2 ratio, of which, five studies reported an improvement(12–15,25) . Notably, the time points of this outcome measurement varied among the studies; for example, Antonaglia et al. (2006) measured PaO_2 immediately prior to and following the first IPV session and also at the time of discharge from ICU, whereas Tsuruta and colleagues (2006) recorded changes in PaO_2 at every 3 hours interval for up to 24 hours(13,14). In contrast, Clini et al. (2006) measured the PaO_2 and PaO_2/FiO_2 ratio at five days interval(12). Despite lack of consistency across the studies, an improvement in oxygenation post IPV

session(s) was found by the majority of the included studies. This improvement in oxygenation could be attributed to the applied positive pressure, which may facilitate gas exchange by increasing overall functional residual capacity(27). Also, positive pressure has been found to unload respiratory muscles which subsequently reduces the oxygen cost of breathing, as demonstrated by Dimassi and colleagues (2011)(26) where the application of IPV led to a 20% reduction in diaphragm loading in post-extubation respiratory failure patients. Some authors also hypothesised that the improvement in oxygenation could, in part, be due to improved airway clearance post IPV intervention(19,21). Three of the included studies in this review reported an observed increase in airway clearance with IPV intervention; however, none of them measured the expectorated sputum weight (wet or dry)(12,13,15).

In addition to improved oxygenation, improved pulmonary ventilation has also been shown to reduce $PaCO_2$ levels in patients with COPD(28,29) when treated with IPV. The applied positive pressure by IPV reduces the airway obstruction and thereby increases the expiratory flow in patients with airflow limitation secondary to COPD(28,29). A study in 25 patients with COPD demonstrated an increase in expiratory flow rate after IPV intervention (25). Studies also noted a reduced $PaCO_2$ levels after application of IPV intervention in patients with COPD and patients with tracheostomy(12,13,15). In contrast, two small size studies were unable to confirm any significant change in $PaCO_2$ levels post IPV intervention(14,26). Based on the available evidence, it appears that IPV may have a role in reducing $PaCO_2$ in hypercapnic respiratory failure patients.

The incidence of pneumonia in critically ill patients is well documented(3,10,30). While there is some evidence that IPV may be effective as an airway clearance modality(18), its role in reducing or preventing the incidence of pneumonia in critically ill patients remain poorly studied. In this review, out of three studies(12,13,20), only one study reported a significant

reduction in the incidence of pneumonia in the IPV group compared to the CPT group(12). Although statistically significant, this small reduction in pneumonia however don’t appear to be clinically meaningful. Surprisingly, the number of cases of pneumonia in all the included studies was very small in both the IPV and control groups. Huynh et al. (2019) reported a total of six (3 in each group) cases of pneumonia in 409 postoperative patients(20). Similarly, Clini et al. (2006) reported only two cases of pneumonia in 46 tracheostomised patients(12). Data pooling and analysis was performed which shows that IPV is not effective in preventing or treating pneumonia in critically ill patients. However due to the small number of studies the results of data analysis are not included in this review but can be accessed (see electronic supplementary material). Due to the small number of studies with very few cases of pneumonia, it is difficult to recommend routine use of IPV in preventing or minimising the incidence of pneumonia in critically ill patients. Further research is needed to evaluate the role of IPV in reducing the incidence of pneumonia.

Clinicians have been using IPV to improve the lung volumes by recruiting partially or fully collapsed lung units(28,29). Despite this, the effect of IPV intervention in preventing or treating pulmonary atelectasis in acutely ill patients remains poorly researched. One reason may be that measuring the changes in pulmonary atelectasis can be challenging and also expensive in a clinical setting. Deakins and Chatburn (2002)(31) used the radiological atelectasis score to assess a series of chest x-rays before and after IPV intervention in pediatric patients, whereas Tsuruta and colleagues (2006)(14) used chest x-rays and computed tomography scans. Huynh et al (2019) reported significant reduction in several post-operative pulmonary complications including atelectasis, however, it is unclear how this change in atelectasis was assessed. In our review, only one study assessed and reported a resolution in compression atelectasis in five out of 7 obese patients when IPV was superimposed on the mechanical ventilator(14). Due to the small sample size of this

observational study, the current level of evidence remains weak and inconclusive regarding the role of IPV in treating pulmonary atelectasis in critically ill patients.

Clinical benefit of an intervention can be influenced by several factors such as adverse effects, poor treatment tolerance and patient compliance. The studies included in this review found IPV intervention to be safe. None of the studies reported any serious adverse events related to IPV intervention. A recent study in 35 critical care patients found that the application of IPV was safe in critical care setting(32). Furthermore, most of the studies in this review also reported that the IPV intervention was well tolerated by patients. However, the studies did not incorporate a subjective measure of tolerance of IPV intervention; instead, this was inferred from observation and treatment completion rates. Only one study, in 17 patients at risk of extubation failure, performed a subjective evaluation of IPV tolerance using a five-point scale ("severe discomfort" = 1 to "very good level of comfort" = 5). An average score of 3 ("acceptable level of comfort") was provided by 16 patients whereas one patient found IPV to be very noisy(26). Further studies are required that incorporate a subjective evaluation of the patient's experience with IPV intervention in critical care.

Limitation

This systematic review has some limitations. The number of studies retrieved was small. While there is a chance that we were unable to find all the relevant studies, we minimised this by searching five databases and six trial registries for the last 40 years of publications. The heterogeneity with study design, patient population, dosage, and frequency of IPV intervention across the included studies affected the strength of conclusions that could be drawn to guide clinicians regarding the implementation of IPV in the critical care setting. The

small sample size in the retrieved studies and lack of control group in some studies also introduces some bias and weakens the strength of conclusion of this review.

Conclusion

This systematic review is the first to summarise the evidence of the IPV intervention in patients admitted to critical care. The findings of this review demonstrate that IPV intervention may have a role in reducing ICU and hospital LOS, reducing respiratory rate, and improving gas exchange in critically ill adult patients. However, this review is based on a small number of available studies mostly with small sample sizes, hence, more clinical trials with larger sample sizes are warranted to further add to the findings of this review.

Acknowledgment

We would like to thank Ms. Kanchana Ekanayake, Academic Liaison Librarian, The University of Sydney, for her assistance with the database search.

Author contribution

Anwar Hassan: Registration, study protocol, search strategy, database search, screening, full-text review, data extraction and synthesis, statistical analysis, and manuscript preparation

William Lai: Study protocol, screening, full-text review, data extraction, and synthesis and manuscript preparation

Prof. Stephen Huang: Study protocol, search strategy, statistical analysis, manuscript preparation, and review

Prof. Jennifer Alison: Study protocol, search strategy, data analysis, manuscript preparation, and review

Dr. Maree Milross: Study protocol, search strategy, screening, full-text review, data extraction, and manuscript preparation and review

References

1. Jelic S, Cunningham JA, Factor P. Clinical review: Airway hygiene in the intensive care unit. Crit Care. 2008;12(2):209.

2. Fialkow L, Farenzena M, Wawrzeniak IC, Brauner JS, Vieira SRR, Vigo A, et al. Mechanical ventilation in patients in the intensive care unit of a general university hospital in southern Brazil: an epidemiological study. Clinics. 2016;71(3):144–51.

3. Kalanuria AA, Zai W, Mirski M. Ventilator-associated pneumonia in the ICU. Crit Care. 2014;18(2):208.

4. Vincent J-L, MD, PhD, Akc¸a FS, Mendonc¸a MA de, Haji-Michael MP, et al. The Epidemiology of Acute Respiratory Failure in Critically Ill Patients. Chest. 2002;(121):1602–9.

5. Franca SA, Toufen C, Hovnanian ALD, Albuquerque ALP, Borges ER, Pizzo VRP, et al. The epidemiology of acute respiratory failure in hospitalized patients: A Brazilian prospective cohort study. J Crit Care. 2011;26(3):330.e1-330.e8.

6. Muscedere JG, Martin CM, Heyland DK. The impact of ventilator-associated pneumonia on the Canadian health care system. J Crit Care. 2008;23(1):5–10.

7. Stefan MS, Shieh M, Pekow PS, Rothberg MB, Steingrub JS, Lagu T, et al. Epidemiology and outcomes of acute respiratory failure in the United States, 2001 to 2009: A national survey. J Hosp Med. 2013;8(2):76–82.

8. Mathai AS, Phillips A, Kaur P, Isaac R. Incidence and attributable costs of ventilator-associated pneumonia (VAP) in a tertiary-level intensive care unit (ICU) in northern India. J Infect Public Heal. 2015;8(2):127–35.

9. Ntoumenopoulos G, Hammond N, Watts NR, Thompson K, Hanlon G, Paratz JD, et al. Secretion clearance strategies in Australian and New Zealand Intensive Care Units. Aust Crit Care. 2018;31(4):191–6.

10. Pozuelo-Carrascosa DP, Torres-Costoso A, Alvarez-Bueno C, Cavero-Redondo I, Muñoz PL, Martínez-Vizcaíno V. Multimodality respiratory physiotherapy reduces mortality but may not prevent ventilator-associated pneumonia or reduce length of stay in the intensive care unit: a systematic review. J Physiother. 2018;64(4):222–8.

11. Skinner EH, Haines KJ, Berney S, Warrillow S, Harrold M, Denehy L. Usual Care Physiotherapy During Acute Hospitalization in Subjects Admitted to the ICU: An Observational Cohort Study. Respir Care. 2015;60(10):1476–85.

12. Clini EM, Antoni FD, Vitacca M, Crisafulli E, Paneroni M, Chezzi-Silva S, et al. Intrapulmonary percussive ventilation in tracheostomized patients: a randomized controlled trial. Intens Care Med. 2006;32(12):1994–2001.

13. Antonaglia V, Lucangelo U, Zin WA, Peratoner A, Simoni LD, Capitanio G, et al. Intrapulmonary percussive ventilation improves the outcome of patients with acute exacerbation of chronic obstructive pulmonary disease using a helmet* Crit Care Med. 2006;34(12):2940–5.

14. Tsuruta R, Kasaoka S, Okabayashi K, Maekawa T. Efficacy and safety of intrapulmonary percussive ventilation superimposed on conventional ventilation in obese patients with compression atelectasis. J Crit Care. 2006;21(4):328–32.

15. Vargas F, Bui HN, Boyer A, Salmi LR, Gbikpi-Benissan G, Guenard H, et al. Intrapulmonary percussive ventilation in acute exacerbations of COPD patients with mild respiratory acidosis: a randomized controlled trial [ISRCTN17802078]. Crit Care. 2005;9(4):R382.

16. Spapen H, Borremans M, Diltoer M, Regt JD, Bruggemans C, Honoré PM. Intrapulmonary percussion with autogenic drainage and ventilator-associated Gram-negative infection: A pilot study. undefined.

17. Lucangelo U, Accardo A, Bernardi A, Ferluga M, Borelli M, Antonaglia V, et al. Gas distribution in a two-compartment model ventilated in high-frequency percussive and pressure-controlled modes. Intens Care Med. 2010;36(12):2125–31.

18. Kallet RH. Adjunct Therapies During Mechanical Ventilation: Airway Clearance Techniques, Therapeutic Aerosols, and Gases. Respir Care. 2013;58(6):1053–73.

19. Reper P, Looy K van. Chest physiotherapy using intrapulmonary percussive ventilation to treat persistent atelectasis in hypoxic patients after smoke inhalation. Burns. 2013;39(1):192–3.

20. Huynh TT, Liesching TN, Cereda M, Lei Y, Frazer MJ, Nahouraii MR, et al. Efficacy of Oscillation and Lung Expansion in Reducing Postoperative Pulmonary Complication. J Am Coll Surgeons. 2019;229(5):458-466.e1.

21. Reychler G, Debier E, Contal O, Audag N. Intrapulmonary Percussive Ventilation as an Airway Clearance Technique in Subjects With Chronic Obstructive Airway Diseases. Respir Care. 2018;63(5):620–31.

22. Moher D, Liberati A, Tetzlaff J, Altman DG, Group TP. Preferred Reporting Items for Systematic Reviews and Meta-Analyses: The PRISMA Statement. Plos Med. 2009;6(7):e1000097.

23. Higgins JPT, Altman DG, Gotzsche PC, Juni P, Moher D, Oxman AD, et al. The Cochrane Collaboration’s tool for assessing risk of bias in randomised trials. Bmj. 2011;343(oct18 2):d5928.

24. Elkins MR, Herbert RD, Moseley AM, Sherrington C, Maher C. INVITED COMMENTARY: Rating the Quality of Trials in Systematic Reviews of Physical Therapy Interventions. Cardiopulm Phys Ther J. 2010;21(3):20–6.

25. Vargas F, Boyer A, Bui HN, Guenard H, Gruson D, Hilbert G. Effect of intrapulmonary percussive ventilation on expiratory flow limitation in chronic obstructive pulmonary disease patients. J Crit Care. 2009;24(2):212–9.

26. Dimassi S, Vargas F, Lyazidi A, Roche-Campo F, Dellamonica J, Brochard L. Intrapulmonary percussive ventilation superimposed on spontaneous breathing: a physiological study in patients at risk for extubation failure. Intens Care Med. 2011;37(8):1269–76.

27. Fornasa E, Ajčević M, Accardo A. Characterization of the mechanical behavior of intrapulmonary percussive ventilation. Physiol Meas. 2013;34(12):1583–92.

28. Dellamonica J, Louis B, Lyazidi A, Vargas F, Brochard L. Intrapulmonary percussive ventilation superimposed on conventional ventilation: bench study of humidity and ventilator behaviour. Intens Care Med. 2008;34(11):2035–43.

29. Nava S, Barbarito N, Piaggi G, Mattia ED, Cirio S. Physiological response to intrapulmonary percussive ventilation in stable COPD patients. Resp Med. 2006;100(9):1526–33.

30. Ntoumenopoulos G, Presneill J, McElholum M, Cade J. Chest physiotherapy for the prevention of ventilator-associated pneumonia. Intens Care Med. 2002;28(7):850–6.

31. Deakins KM, Chatburn RL. A comparison of intrapulmonary percussive ventilation and conventional chest physiotherapy for the treatment of atelectasis in the pediatric patient. Respiratory care. 2002; 47: 1162-7.

32. Hassan A, Milross M, Lai W, Shetty D, Alison J, Huang S. Feasibility and safety of intrapulmonary percussive ventilation in spontaneously breathing, non-ventilated patients in critical care: A retrospective pilot study. J Intensive Care Soc. 2020;175114372090970.

Figure 1 PRISMA Flow diagram

Click here to access/download;Figure;Figure 1_prisma_A_Hassan.docx

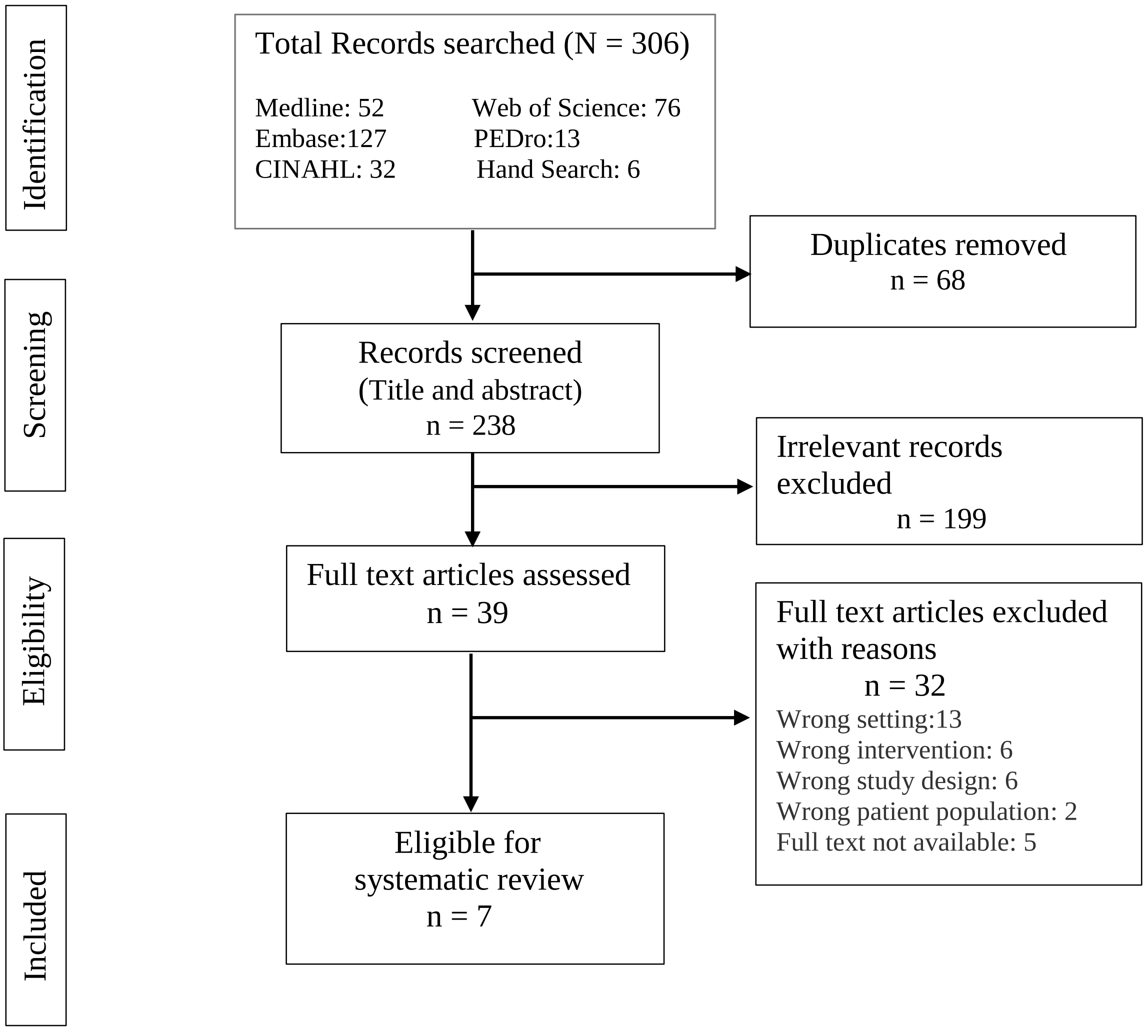

Figure 1. PRISMA flow chart

Table 2. Study summary

Author (Year)	Study design, N	Population	Inclusion and exclusion criteria	Intervention(s) (Setting)	Outcomes
**Antonaglia et al. (2006)**	RCT<br><br>N = 80<br><br>IPV = 20<br>CPT = 20<br>Control = 40	Acute exacerbation of COPD<br><br>Age: 69.75±6.56 years<br>Gender: Not reported	**Inclusion**<br>admitted to ICU with COPD<br>RR >25 bpm<br>pH: 7.10–7.35<br>$PaCO_2$ >50 mm Hg<br><br>**Exclusion**<br>GCS < 8<br>> two organ failure<br>Hemodynamic instability<br>Requirement for sudden intubation	**IPV group**<br>Duration: 30 min (via mouthpiece) twice a day<br>IPV Setting: 225 cycles / min, P_{AW} < 40 cmH_2O<br><br>**Control group**<br>Standard medical care<br><br>**CPT group**<br>Duration: 30 min CPT (chest clapping, mobilisation, and postural drainage, and expiration with the glottis open) twice a day	**Gas exchange**<br>After the 1st IPV session<br>IPV: ↑ pH**, ↓ $PaCO_2$**, ↑ PaO_2/FiO_2**<br>At discharge:<br>IPV: pH (NS), ↓ $PaCO_2$**, ↑ PaO_2/FiO_2**<br>Control: ↓ $PaCO_2$**, ↑ PaO_2/FiO_2**<br>**Cardio-respiratory parameters**<br>After the 1st session:<br>IPV: ↓ RR*, ↓ HR*<br>**ICU-LOS** (days)<br>IPV: 7 [6-8], Control:10 [9-11]**<br>**Pneumonia**<br>IPV: 2 vs. Control: 11(NS)
**Vargas et al. (2005)**	RCT<br><br>N = 33<br><br>IPV: 16<br>Control: 17	Patients admitted to ICU with exacerbation of COPD<br><br>Age = 69.71±5.44 years<br>Gender: Not reported	**Inclusion**<br>Exacerbation of COPD<br>RR ≥ 25/min<br>$PaCO_2$ > 45 mmHg<br>pH: 7.35 to 7.38<br><br>**Exclusion**<br>Need for intubation<br>GCS ≤ 8<br>Hemodynamic instability<br>> two organ failure<br>Tracheostomy<br>Pneumothorax, oral/oesophageal/gastric surgery	**IPV group**<br>Duration: 30 min (via a face mask), twice a day<br>Setting: 80-650 cycles / min, P_{AW}: 5-35cmH_2O<br>IPV sessions were stopped when a RR of < 25/min and a pH > 7.38 was reached<br><br>**Control group**<br>Standard medical care<br>Oxygen to maintain SpO_2 of 88% to 92%<br>HOB elevated at a 45-degree angle<br>Nebulised bronchodilators and corticosteroids	**Gas exchange**<br>IPV: ↑ PaO_2*, ↓ $PaCO_2$*<br>Control: Not reported<br>**Cardio-respiratory parameters**<br>IPV: ↓ RR*, Control: Not reported<br>**ICU-LOS** (days)<br>IPV: 6.8±1.0, Control: 7.9±1.3*<br>**Need for NIV**<br>IPV: (0) 0%, Control: (6) 35.3%*
**Dimassi et al. (2011)**	Randomised (crossover) to receive IPV or NIV<br><br>N = 17<br><br>IPV: 8<br>NIV: 9	ICU patients at risk for post-extubation failure<br><br>Age: 73 [58-75] years<br>Gender = M 14, F 3	**Inclusion**<br>Intubation > 48 h<br>Age > 65<br>underlying heart or respiratory failure, and<br>APACHE II score > 12<br><br>**Exclusion**<br>Tracheostomy<br>Facial or cranial trauma or surgery<br>Recent gastric/oesophageal surgery<br>Active UGI bleeding<br>Lack of cooperation<br>Limit of therapy in ICU	**IPV group**<br>Duration: 20 minutes (via a face mask)<br>Setting: 250 cycles / min, P_{AW}: 1.2 bar, I:E: 1 / 2.5<br><br>**Control group (NIV)**<br>Settings: Delivered via IVU ventilator in pressure-support mode with PEEP. Tidal volume target 6-8ml/kg, PEEP 4-5 cmH_2O	**Gas exchange**<br>IPV: $PaCO_2$ (NS), PaO_2/FiO_2 (NS)<br>Control (NIV): ↓$PaCO_2$**, PaO_2/FiO_2 (NS)<br>**Cardio-respiratory parameters**<br>IPV: ↓RR**, Control: ↓RR**<br>**Diaphragmatic work (PTPdi/breath)**<br>IPV: ↓20%, Control: ↓35% (NS)

**Clini et al. (2006)**	RCT<br><br>N = 46<br><br>IPV group: 24<br>CPT group: 22	Tracheostomised patients randomised to two treatment groups<br><br>Age: 68.96±9.06 years<br>Gender = M 28, F 18	**Inclusion**<br>Mechanically ventilated ≥ 14 days<br>Passed the SBT for at least 72 h<br>Sputum > 40ml/day<br><br>**Exclusion**<br>Haemodynamic instability<br>Reconnection to ventilator < 72 hours<br>Need for continuous sedatives and vasopressors	**IPV group**<br>IPV + CPT twice / day (via tracheostomy tube)<br>Duration: 10 min<br>Setting: 200 – 300 cycles / min, P_{AW}: 40cm H_2O, I/E: 1:1.2<br><br>**Control group**<br>CPT for 1-hr, twice / day	**Gas exchange**<br>IPV group vs. Control group during the treatment period: ↓ pH (NS), ↑ PaO_2 (NS), ↑ PaO_2/FiO_2*, ↓ $PaCO_2$ (NS)<br>**Cardiorespiratory parameters**<br>IPV group vs. Control group during the treatment period: ↑ MEP*<br>**Pneumonia** (at one month follow up)<br>IPV: 0, CPT: 2*
**Huynh et al. (2019)**	Multicentre prospective observational study<br><br>N = 419<br><br>IPV: n = 209<br>CPT: n = 210	Post-thoracic, upper abdominal and aortic surgery patients admitted to ICU<br><br>Age: 59.25±14.73 years<br>Gender: M 246, F 173	**Inclusion**<br>Age ≥ 18 years with thoracic, upper abdominal and aortic surgery<br>ASA class ≥ 3 OR 2 with one or more of the following: current smoker; COPD; BMI ≥ 30<br><br>**Exclusion**<br>Contraindication to positive pressure therapy, organ transplant, spinal surgery, and positive pressure ventilation at baseline.	**IPV group**<br>Received IPV in addition to standard care<br>Duration: 10 minutes per session<br>IPV for intubated patients x 6 / day<br>Non-intubated patients x 4 / day<br><br>**Control group**<br>Standard care including incentive spirometer. Additional respiratory treatment was provided based on clinical indication	**Gas exchange** Not measured<br>**Cardiorespiratory parameters** Not measured<br>**ICU-LOS** (days)<br>IPV: 3.4±3.5, CPT 5.4±8.7 (NS)<br>**Time on mechanical ventilation**<br>IPV: 29.7±44.8 hours to 94.1±199.2*<br>**Pneumonia:** no difference<br>**Hospital LOS**<br>IPV: 6.78±4.5, Control: 8.4±7.9* days
**Tsuruta et al. (2006)**	Prospective observational study<br><br>N =10<br>Control: not assigned	Mechanically ventilated patients with compression atelectasis<br><br>Age: 52±19 years<br>Gender: M 8, F 2<br>BMI: 31±6	**Inclusion**<br>Acute respiratory failure due to atelectasis unresolved by conventional ventilation<br>BMI > 25<br><br>**Exclusion**<br>Infiltrations induced by infection and drugs	**IPV group**<br>IPV delivered via in-line ventilator circuit<br>Duration and frequency: Not reported<br>Setting: 300 cycles/min	**Gas exchange**<br>Pre IPV compared to 24h post IPV<br>PaO_2/FiO_2 189±63 to 280±55*<br>$PaCO_2$: 38 to 37 (NS)<br>**Cardiorespiratory parameters: NS**<br>**Improvement of atelectasis**<br>7 out of 10 patients improved on chest CT scans
**Vargas et al. (2009)**	Prospective observational study<br><br>N = 25<br><br>IPV: n = 25<br>Control: not assigned	COPD patients with expiratory flow limitation were screened following extubation<br><br>Age: 63±8 years<br>Gender: M 15, F 10<br>BMI: 26±3	**Inclusion**<br>Diagnosis of COPD<br>RR < 30/min<br>Lack of respiratory acidosis with a pH > 7.35<br><br>**Exclusion**<br>Need for intubation<br>GCS ≤ 8<br>Hemodynamic instability<br>> two organ failure<br>Tracheostomy<br>Pneumothorax, oral/oesophageal/gastric surgery	**IPV group**<br>Device: IPV (via a full-face mask)<br>Duration: 30 minutes<br>Settings: 250 cycles / min, P_{AW}: 20cmH_2O<br><br>Oxygen was interfaced into the mask to achieve target oxygen saturation between 88% and 92%.	**Gas exchange**<br>↑ pH: 7.39±0.01 to 7.4±0.01*<br>↑ PaO_2: 8.3±0.4 to 8.8±0.4 (kPa)*<br>↓ $PaCO_2$: 7.35±0.5 to 7.0±0.3 (kPa)*<br>↑ SpO_2: 90±2% to 93±3%*<br><br>**Cardio-respiratory parameters**<br>↓ HR (5.3%)*, ↓ RR: 22.6±2.3 to 21.4±1.7*<br>Expiratory flow limitation: ↓ 31%*<br>Airway occlusion pressure: ↓ 28.2%*

RCT = randomised controlled trial, IPV = intrapulmonary percussive ventilation, COPD = chronic obstructive pulmonary disease, CPT = chest physiotherapy, SBT = spontaneous breathing trial, RR = respiratory rate, HR = heart rate, MAP = mean arterial pressure, P_{AW} = airway pressure, MEP = maximal expiratory pressure, HOB = head of bed, I:E = inspiratory to expiratory cycle ratio, APACHE II = Acute Physiology and Chronic Health Evaluation II, PPC = postoperative pulmonary complication, UGI = upper gastrointestinal, NIV = non-invasive ventilation, PTPdi = diaphragmatic pressure-time product, V_T = tidal volume, MEP = maximal expiratory pressure, ASA = American Society of Anesthesiologists, BMI = body mass index, NS = not significant, * = statistically significant $p \leq 0.05$, ** = statistically

Click here to access/download
Supporting Information
Supplementary material_IPV_Meta-Analysis.docx

Search strategy, supplementary table 1s

Click here to access/download
Supporting Information
Supplemenatry table
1(S)_search_strategy_A_Hassan.docx

PRISMA Checklist

Click here to access/download
Supporting Information
PRISMA_checklist.docx
